# Additional Members to the State Medical Society

**Published:** 1855-09

**Authors:** 


☞ By reference to the proceedings of the State Medical Society, we find that there has been an omission of several names who were elected members of the Society, viz: Drs. J. C. Bennett, Henry C. Grimmell, Thomas K. Brooks, William P. Davis and Smith Y. Campbell. J. C. Hanson, M. D., of Great Falls, New Hampshire, was elected Honorary Member, with all the rights and priviliges as such.

